# Cool Down–Warm Up: Differential Responses of Stored Product Insects after Gradual Temperature Changes

**DOI:** 10.3390/insects11030158

**Published:** 2020-03-01

**Authors:** Christos G. Athanassiou, Frank H. Arthur

**Affiliations:** 1 Laboratory of Entomology and Agricultural Zoology, Department of Agriculture, Crop Production and Rural Environment, University of Thessaly, Phytokou Str., Nea Ionia 38446, Magnissia, Greece; 2 Center for Grain and Animal Health Research, Agricultural Research Service, United States Department of Agriculture (USDA), 1515 College Avenue, Manhattan, KS 66502, USA; Frank.Arthur@ARS.USDA.GOV

**Keywords:** cold treatments, acclimation, stored-product beetles, non-chemical control

## Abstract

Insect survival after exposure to 0 °C for 7 days was examined in laboratory bioassays for control of adults of six major stored-product beetle species, *Oryzaephilus surinamensis* (L.), the sawtoothed grain beetle, *Cryptolestes ferrugineus*, (Stephens), the rusty grain beetle, *Dermestes maculatus* DeGeer, the hide beetle, *Sitophilus oryzae* (L.), the rice weevil, *Tribolium castaneum* (Herbst), the red flour beetle, and *T. confusum* Jacquelin DuVal, the confused flour beetle In this test there were four different acclimation treatments, insects that had been subjected to a pre-acclimation period to 0 °C, a post-acclimation period, both a pre and post-acclimation period, and adults that were not acclimated. Insect survival for all species except *S. oryzae* was not affected by the exposure to 0 °C, regardless of the acclimation scenario. In contrast, exposure to 0 °C drastically reduced survival of *S. oryzae*. Moreover, adults that were exposed to the post-acclimation only and un-acclimated adults had lower survival rates than those that had either exposure to pre-acclimation, or to both pre- and post-acclimation. Results of this experiment show that acclimation played a limited role in adult survival of five of the six tested species, and that exposure of adults to 0 °C for 7 d had no effect in survival of these species as well.

## 1. Introduction

The application of low temperatures for control of stored-product insects has been extensively evaluated over the past three decades for many species of the orders Coleoptera, Lepidoptera and Psocoptera [1,2,3,4,5,6,7,8]. These studies show that stored-product insects vary remarkably in their susceptibility to low temperatures. For example, Athanassiou et al. [6] found that mortality of eggs of *Plodia interpunctella* (Hübner), the Indian meal moth (Lepidoptera: Pyralidae), was 100% after 7 d and 2 d at 0 and −5 °C, respectively. In contrast, the same study showed that eggs of the psocid *Liposcelis bostrychophila* Badonnel (Psocoptera: Liposcelididae) could survive for 3 d when exposed to −15 °C [6]. These tests clearly suggest that cold treatments should be carefully designed for a given target species rather than relying on generic recommendations of specific exposure and temperature combinations.

The majority of existing studies focus on the effect of temperatures below 0 °C. This is partially due to the fact that many major stored-product insect species can survive for several days, or even weeks, when exposed to 0 °C [1,4,7,9]. Exposure to temperatures lower than 0 °C may shorten the time required for mortality, but these lower temperatures may require more energy and thus may not be economically feasible in some instances. The effect of extreme low temperatures, such as −18 °C (=0 °F), has also been evaluated, with recent studies suggesting that, in many cases, subzero temperatures of −5 °C are equally as effective as subzero temperatures of −18 °C [6,7,8]. This underlines the potential of operating cold treatments successfully at temperatures that are closer to 0 °C than to −18 °C. Athanassiou et al. [6] found that approximately 90% of eggs of *P. interpunctella* were dead after exposure to −5 °C for only 8 h.

Acclimation is considered to be a key element in cold tolerance of insects but has not been examined in detail in the case of stored-product insects, despite the fact that these insects are good candidates for the exploitation of “real world” cold treatments. A recent review by Andreadis and Athanassiou [4] underlines the lack of paradigms of cold tolerance of stored-product insects as compared with field crop pests. In addition, most of the data available for stored-product insects’cold tolerance are largely focused on non-acclimated individuals [1,3,5,7,9]. Athanassiou et al. [7] show that acclimation of stored-product insects prior to actual treatment has dissimilar effects depending on species and acclimation conditions, and is not always related to increased tolerance. In the study, the authors tested two major stored-product beetle species, *Oryzaephilus surinamensis* (F.), the saw-toothed grain beetle (Coleoptera: Silvanidae), and *Tribolium confusum* Jacquelin du Val (Coleoptera: Tenebrionidae), the confused flour beetle, and found that acclimation before exposure to subzero temperatures, in many of the combinations tested, led to increased mortality instead of decreased mortality. These results show that certain species and life stages may be negatively affected by acclimation and this acclimation may lead to quicker death when exposed to sub-zero temperatures compared to non-acclimated individuals. Thus, acclimation may not necessarily lead to cold hardiness but may instead accelerate death. In this context, some stored-product insect species may be opportunistic in terms of cold tolerance [4,8].

Acclimation studies are almost exclusively based on a “cool down” approach, a gradual or abrupt exposure to low temperatures before the cold treatment [1,4,8,10]. In this scenario, insects are “cooled down” to temperatures that are low but not lethal, and the susceptibility of these insects is usually compared with that of non-acclimated individuals. Theoretically, previous exposure allows insects to minimize their key physiological functions, and produce the so-called “cryoprotectants” that will eventually lead to cold hardiness [4]. In most studies, mortality is examined either right after the termination of the exposure, or after a certain period of post-exposure at normal temperatures, especially in the case of eggs and pupae, for which mortality cannot be evaluated immediately after the exposure. In contrast, there are no data currently available for the effect of a “warm up” approach, which is not based on pre-conditioning of the insects that are to be exposed, but to a post-conditioning of the insects that have already been exposed. Like pre-acclimation, the post-acclimation of insects may be critical for the efficacy of cold treatments and could provide a gradual increase of physiological functionality as compared with insects that are directly exposed to elevated temperatures right after the moment that the cold treatment is terminated. Still, this post-acclimation approach has not been tested in detail, either as a stand-alone scenario or in conjunction with pre-acclimation. Practically, post-acclimation is very likely to occur when durable commodities are treated with subzero temperatures, as temperature increases very gradually after the termination of the cold treatments [5], which may allow insects to have a considerable adjustment period. Hence, in these scenarios, insect mortality may not be solely caused by the cold treatment itself, but by a combination of cold treatment with post-acclimation.

The concept of cold-tolerance needs to be more fully developed for stored-product insects. Additional data on adult beetles could help explain some of the recent results in the scientific literature. The objective of our study was to examine this “cool down–warm up” scenario of insect exposure to 0 °C for six stored-product beetle species. The research was conducted using combinations of presence and absence of pre and post-acclimation, applied either alone or together, to provide additional data for further exploitation of cold treatments in realistic conditions of gradual temperature changes.

## 2. Materials and Methods

### 2.1. Insects

The stored-product beetle species tested were: *O. surinamensis*, the rusty grain beetle; *Cryptolestes ferrugineus* (Stephens) (Laemophloeidae), the hide beetle; *Dermestes maculatus* (DeGeer) (Dermestidae), the rice weevil; *Sitophilus oryzae* (L.) (Curculionidae), the red flour beetle; *Tribolium castaneum* (Herbst) (Tenebrionidae); and *T. confusum*. All insects were taken from standard laboratory cultures maintained at the Center for Grain and Animal Health Research (CGAHR), United States Department of Agriculture, Agricultural Research Service (USDA-ARS), Manhattan, KS, USA, where they were kept at 27.5 °C and 70% relative humidity (RH). Rearing diets were as mentioned in previous studies [11,12]. For all species, only adults less than two weeks old were used in the bioassays. All species other than *D. maculatus* had been in culture for more than 30 years. *Dermestes maculatus* had been in culture for about 5 years.

### 2.2. Bioassays

All tests were carried out in an incubator (Percival I36NLXC9, Percival Scientific, Perry, IA, USA), where temperatures could be controlled [3]. Plastic cylindrical vials of 50 mm in height and 25 mm in diameter (Thornton Plastics, Salt Lake City, UT, USA) were used in the tests. There were 10 adults within each vial, with different vials for each species. Each vial contained about 50 mg of food, i.e., oats for *O. surinamensis*, cracked wheat kernels for *C. ferrugineus*, dog food for *D. maculatus*, whole wheat kernels for *S. oryzae* and wheat flour for *T. castaneum* and *T. confusum*. All vials were placed in the incubator which was initially set at 25 °C, then adjusted after 7 d to 0 °C, maintained at 0 °C for 7 d and then increased again to 25 °C. Thus, the total duration of the treatment was 21 d. In this context, we had five different treatment scenarios: (a) insects that remained at 25 °C for the entire 21-d period (control); (b) insects that remained at 25 °C for 7 d, at 0 °C for 7 d and then at 25 °C for 7 d , without any gradual change of the temperature (non-acclimated); (c) insects exposed to a gradual decrease from 25 to 0 °C for 7 d, then kept at 0 °C for 7 d before being taken back to 25 °C for 7 d without a gradual increase (pre-acclimated only); (d) insects that remained at 25 °C for 7 d, then, without a gradual decrease from 25 to 0 °C, kept at 0 °C for 7 d, before going through a gradual increase from 0 to 25 °C for 7 d (post-acclimated only); and e) insects exposed to a gradual decrease from 25 to 0 °C for 7 d, kept at 0 °C for 7 d then exposed to a gradual increase from 0 to 25 °C for 7 d (both pre and post-acclimated). After the termination of this 21-d period, all vials were opened, and adult survival was recorded. There were three vials for each combination, while the entire procedure was repeated three times (three blocks of three replicates, equaling nine vials in total per species and treatment).

### 2.3. Data analysis

The data were analyzed separately for each species. Before analysis, all measurements were tested for unequal variances with O’Brien tests, which indicated that no transformation was necessary (see footnote of Table 1). Then, the data were analyzed using a one-way Analysis of variance (ANOVA) for each species, with treatment as the main effect and insect survival as the response variable. Means were separated by using the Tukey-Kramer HSD test at *p* = 0.05.

## 3. Results

Four out of the six examined species showed no significant differences among the exposure treatments (Table 1). There was little mortality of *O. surinamensis* and *T. castaneum*, as most adults were still alive after the 21-day trial, regardless of the acclimation treatment. No significant differences were noted between the adults that had been exposed to 0 °C and the control adults (Table 2). Similarly, for *C. ferrugineus* and *D. maculatus*, there were no significant differences among treatments, but there was some mortality in certain combinations. This was expressed more vigorously for *C. ferrugineus*, where survival was always lower in the insects that had undergone the exposure scenario as compared with the controls. This was especially noticeable for the post-acclimated adults.

*Sitophilus oryzae* was the most susceptible species tested here (Table 2). Moreover, for this species, there were significant differences in adult survival among treatments. Survival of *S. oryzae* was much lower in the exposure treatments as compared to the controls, and ranged between 27% and 69%. The lowest survival rates were noted for non-acclimated adults and adults that were only post-acclimated, which was significantly lower than that in the only pre-acclimated adults and adults that were both pre and post-acclimated. Similarly, for *T. confusum*, there were significant differences among treatments but survival was much higher than that of *S. oryzae*, and, in all combinations, exceeded 92% (Table 2). For this species, survival of adults that were post-acclimated only was significantly lower than that of the control adults or the non-acclimated adults. For these last two treatment categories, all exposed adults were alive after the 21-d interval.

## 4. Discussion

Our results show that pre and post-acclimation may play a role in insect survival when exposed to cold, but this role is expressed only in certain species. Despite the fact that there were some differences for *T. castaneum*, these can be considered rather marginal, given that survival was still high even for the adults that had been affected by the treatment. In an earlier study, Athanassiou et al. [8] tested different scenarios of acclimated and non-acclimated life stages of *O. surinamensis* and *T. confusum*, at temperatures ranging from 0 and −15 °C, and described differential effects of acclimation for both species. For example, for *T. confusum*, non-acclimated pupae were as equally susceptible as acclimated ones, but in many other combinations of exposures, temperatures and life stages, acclimated individuals were more cold-tolerant than non-acclimated ones. In contrast, for *O. surinamensis* at 0 °C, non-acclimated larvae had higher survival rates than acclimated ones, but this was reversed at −5 °C. Based on this, acclimation may not be that critical and consistent in terms of the potential increases in cold tolerance. In the study cited above, the authors followed another acclimation approach, as all individuals were acclimated at a constant temperature (+15 °C) for 7 d prior to exposure to cold [8]. In our study, pre and post-acclimation periods were much more gradual, which might have allowed for some adjustment of the exposed adults to cold. This is particularly important as gradual temperature changes may provide the time that is necessary for the insects to trigger cold hardiness mechanisms [4].

*Sitophilus oryzae* was the most susceptible species tested in our study, and, generally, any combination of acclimation increased mortality compared with the controls. Our data are in accordance with previous studies that indicate susceptibility of this species to cold [1,9]. Post-application alone produced the greatest mortality. Post-acclimation might have increased the cold stress by practically prolonging the exposure interval to low temperatures, and most adults were not able to recover. Interestingly, adults that were only post-acclimated had a similar survival rate to non-acclimated adults, which can be considered to be an indication that pre-acclimation may be more critical in cold tolerance increase than post-acclimation. Similar to *S. oryzae*, the lowest survival of *T. confusum* adults was noted in the adults that were only post-acclimated.

The fact that we carried out our tests at 0 °C might conceal the actual effect of acclimation as this is expected to be expressed more vigorously at temperatures that are far lower than 0 °C. For *O. surinamensis* and *T. confusum*, acclimation resulted eventually in increased cold tolerance at −5 °C or lower, especially with longer exposure intervals that exceeded 2 d [8]. In our study, the tested acclimation conditions played a limited role, as, in the majority of combinations, adult mortality was not drastically affected. With the exception of *S. oryzae*, where cold treatment did affect adult survival, exposure of the other species to 0 °C had little or no effect, irrespective of the acclimation scenario. The binary acclimation scenario evaluated here may be realistic in terms of commercial applications, such as gradual seasonal conditional changes or chilling/aeration of the commodity. The exposure of beetle adults to 0 °C was found to be ineffective for most species, but post-acclimation partially differentiated the results in terms of cold tolerance. The post-acclimation effects merit additional investigation in a combined approach with the pre-acclimation, under a “cool down–warm up” approach that can be expanded in low-temperature treatments.

## 5. Conclusions

The results of our study suggest that exposure of stored-product beetle adults to 0 °C does not cause any considerable mortality and thus subzero temperatures should be further investigated towards this end. Moreover, even in the case of *S. oryzae*, adult survival after exposure to 0 °C was high, which renders this application ineffective. At the same time, acclimation played some marginal role, but far less than the acclimation effects when insects are exposed to temperatures that are lower than 0 °C. Our study examined a cool down–warm up approach, demonstrating the importance of a post-exposure acclimation in insect survival.

## Figures and Tables

**Table 1:** Analysis of variance (ANOVA) parameters for different treatments of acclimated and non-acclimated adults * of six stored-product insect species (in all cases, df = 4.40, treatments were: control, non-acclimated, only pre-acclimated, only post-acclimated and both pre and post-acclimated adults) *.

Species	*F*	*p*
*Oryzaephilus surinamensis*	0.1	0.98
*Cryptolestes ferrugineus*	0.6	0.67
*Dermestes maculatus*	1.8	0.16
*Sitophilus oryzae*	16.5	<0.01
*Tribolium castaneum*	0.8	0.55
*Tribolium confusum*	3.8	0.01

* Un-equal variance tests (O’Brien test): for *Oryzaephilus surinamensis*: F = 0.3, *p* = 0.88; for *Cryptolestes ferrugineus*: F = 2.3, *p* = 0.08; for *Dermestes maculatus*: F = 2.0, F = 0.11; for *Sitophilus oryzae*: F = 2.5, *p* = 0.06; for *Tribolium castaneum*: F = 1.2, F = 0.31; for *Tribolium confusum*: F = 1.9, *p* = 0.13; in all cases df = 4.40.

**Table 2:** Mean adult survival (100 ± standard error (SE)) of six different stored-product beetle species after exposure to 0 °C for 7 d, with different pre and post-exposure temperatures. Pre-acclimation indicates a gradual 7d decrease of the temperature from 25 to 0 °C, post-acclimation indicates a gradual 7 d increase of the temperature from 0 to 25 °C, control indicates that the insects were continuously kept at 25 °C (within each row, means followed by the same lowercase letter are not significantly different; where no letters exist, no significant differences were noted; HSD at 0.05).

Species/Conditions *	Control	No Acclimation	Only Pre-Acclimation	Only Post-Acclimation	Both Pre and Post-Acclimation
*O. surinamensis*	97.8 ± 1.4	97.8 ± 1.7	97.8 ± 2.2	96.7 ± 2.4	96.7 ± 2.2
*C. ferrugineus*	92.2 ± 3.2	87.8 ± 4.0	86.7 ± 5.5	81.1 ± 7.0	87.8 ± 5.0
*D. maculatus*	100 ± 0.0	96.7 ± 1.7	98.9 ± 1.1	98.9 ± 1.1	100 ± 0.0
*S. oryzae*	94.4 ± 2.9 a	33.3 ± 6.7 c	67.8 ± 6.2 b	26.7 ± 7.1 c	57.8 ± 9.2 b
*T. castaneum*	100 ± 0.0	97.8 ± 2.5	95.6 ± 2.4	95.6 ± 2.9	96.7 ± 2.4
*T. confusum*	100 ± 0.0 a	100 ± 0.0 a	97.8 ± 1.5 ab	92.2 ± 3.2 b	98.9 ± 1.1 ab
